# Smoking among immigrant groups in metropolitan France: prevalence levels, male-to-female ratios and educational gradients

**DOI:** 10.1186/s12889-018-5379-8

**Published:** 2018-04-11

**Authors:** Myriam Khlat, Damien Bricard, Stéphane Legleye

**Affiliations:** 1 Institut National d’Etudes Démographiques, 133 boulevard Davout, 75980 Paris Cedex 20, France; 2 Institut de Recherche et Documentation en Economie de la Santé, Paris, France; 3 Institut National de la Statistique et des Etudes Economiques (Insee), Montrouge, France; 4 CESP, Faculté de médecine, Université Paris Sud; Faculté de médecine UVSQ; Inserm; Université Paris Saclay, 12 avenue Paul Vaillant Couturier, 94800 Villejuif, France

**Keywords:** Smoking, Cigarette epidemic, Smoking transition, Unhealthy assimilation, Immigrants, France

## Abstract

**Background:**

Although the French population comprises large and diverse immigrant groups, there is little research on smoking disparities by geographical origin. The aim of this study is to investigate in this country smoking among immigrants born in either north Africa, sub-Saharan Africa or French overseas *départements*.

**Methods:**

The data originate from the 2010 Health Barometer survey representative of metropolitan France. The subsample of 20,211 individuals aged 18–70 years (born either in metropolitan France or in the above-mentioned geographical regions) was analysed using logistic regression.

**Results:**

Both immigrants from sub-Saharan Africa and immigrants from overseas *départements* were protected from smoking compared to the reference population, and the former had a distinctive strongly reversed educational gradient in both genders. Returned former settlers from the French colonies in North Africa (repatriates) had the highest smoking levels. Natives from the Maghreb (Maghrebins) showed considerable gender discordance, with men having both a higher prevalence (borderline significance) and a reversed gradient and women having lower prevalence than the reference population.

**Conclusion:**

Immigrants from regions of the world in stage 1 of the cigarette epidemic had relatively low smoking levels and those from regions in stage 2 had relatively high smoking levels. Some groups had a profile characteristic of late phases of the cigarette epidemic, and others, some of which long-standing residents, seemed to be positioned at its early stages. The situation for Maghrebins reflected the enduring influence of gendered norms post-migration. Based on their educational gradients, immigrants from overseas *départements* (particularly men) and Maghrebin women may be at risk of losing their particularly low prevalence. Immigrants from sub-Saharan Africa could retain it. In-depth analysis of smoking profiles of immigrants’ groups is essential for a better targeting of smoking prevention and cessation programs.

## Background

Smoking is considered a major health risk for disadvantaged groups, and one of the most important determinant of social inequalities in health and mortality in the developed world [1]. As immigrants have overall a disadvantaged socioeconomic profile, they are disproportionately represented within the population subgroups which are more concerned by smoking. And yet, they seem to escape this social determinism in certain contexts, such as the USA. In this country, there is evidence in favour of a protective effect of immigrant status against smoking [2–4], to the point that differences in smoking have even been considered as the major cause of America’s migrant mortality advantage [5]. Some authors have called for future research to investigate the variation across migrant groups of the protective effects of foreign-born status [2]. Also, it has been argued that ignorance of immigrant status in the presentation of national-level statistics may obscure large disparities of relevance for the development of public health prevention programs [4].

In a recent review of the literature on smoking in immigrants, a majority of the studies retrieved were from the USA, and only 11 out of 27 had stratified on gender [6]. In Europe, studies of smoking in immigrants are scarce, and mostly concentrated in Germany [7–9] and the Netherlands [8, 10, 11], with little recent research in France [12, 13]. And yet, the French context is of particular interest, as this country has reached the most advanced stage of the cigarette epidemic, with a sedimentation of smoking in the socially disadvantaged groups [14, 15]. Further to that, France constitutes an excellent background for exploring smoking behaviour and disparities, due its distinctive population composition, characterized by the importance and diversity of the immigrant groups in presence.

Our study objective is to describe and contrast in metropolitan France the cigarette smoking patterns of the largest immigrant groups, which originate from countries or regions of the world less advanced in the course of the cigarette epidemic: North Africa, sub-Saharan Africa and French overseas *départements*. A comprehensive analytical approach was adopted by jointly considering the following dimensions: the prevalence levels in immigrants relative to the reference population, their men-to-women prevalence ratios and their educational gradient in prevalence. In addition, an original comparative perspective was developed by covering both those who were born French either in the former colonies in North Africa (repatriates) or in overseas *départements*, and those who were born abroad with a foreign nationality. Our analysis and interpretation builds on the *health behaviour and acculturation hypotheses* [16, 17] and the *cigarette epidemic* [18, 19] *and smoking transition models* [6, 10], with particular attention to the difference in stages of the epidemic reached in the countries of origin of the immigrants and in France.

## Method

### Data

The 2010 ‘Health Barometer’ is a nationwide French survey that used a two-stage random sampling frame (household/individual) to measure health perceptions and behaviours of the general population [20]. This investigation was approved by the French Commission on Individual Data Protection and Public Liberties (the *Commission Nationale Informatique et Libertés (CNIL)*), and all data collected were anonymous and self-reported. The response rate was 61%. Survey weights were obtained through a calibration procedure considering phone type, age, educational level and region of residence to match the distribution of the last national Labor Force Survey. The initial sample of respondents comprised 27,653 individuals aged 15–85 years having answered questions about their migration and smoking histories. Our analyses concerned the subsample of 20,211 individuals aged 18–70 years and born either in metropolitan France or in any of the geographical regions of interest. The detailed methodology can be found in [21].

### Measures

The outcome is the daily tobacco smoking, defined as a current smoking of tobacco (whatever the type of product), excluding the electronic cigarette. Immigrants were categorized based on six items of information: place of birth and nationality at birth and mothers’s and father’s place of birth and nationality. Due to confidentiality and legal data protection constraints, the places of birth were collected during interview but immediately aggregated into large regions of birth. Given this loss of information, immigrants belonging to the oldest waves from southern Europe could not be separated from those originating from other European countries, and those from south-east Asia were not large enough to constitute a distinct group. Immigrants from North Africa were subdivided into repatriates (French nationals born in the former colonies of North Africa) and natives from those colonies born without the French nationality, who are referred to hereinafter as the Maghrebins. The latter term (Maghrebins) is derived from an Arabic term for the North African region of origin, to refer to immigrants and their descendants who have neither European backgrounds nor Jewish origins [22]. The analysis was limited to the following: reference group (born French in metropolitan France from parents born in metropolitan France); born in overseas *départements;* Maghrebins; born in sub-Saharan Africa and; repatriates from the Maghreb.

**Table 1:** Description of the study sample according to background characteristics (ages 18–70 years)

	Unweighted	Weighted					
	Absolute number	Median age at survey	Median age at arrival in metropolitan France	Median duration of stay in France	% Low education^(a)^	% High education^(b)^	Percentage of daily smokers
Men							
Reference group	8407	43			23.9	13.0	32.5
Migrants group							
Maghrebins	251	40	20	20	41.2	11.8	41.1
Repatriates from north Africa	146	57	9	48	35.6	20.3	33.8
Migrants from sub-Saharan Africa	147	39	22	15	34.1	18.5	18.7
Migrants from overseas french *départements*	137	38	18	24	34.9	7.8	21.5
Women							
Reference group	10,356	44			28.8	13.5	26.5
Migrants group							
Maghrebins	221	40	18	24	51.6	10.9	13.6
Repatriates from north Africa	181	58	10	48	37.5	13.9	21.5
Migrants from sub-Saharan Africa	196	34	19	13	41.0	12.7	18.8
Migrants from French overseas *départements*	169	38	19	20	24.2	9.3	14.8

^a^Low education: below upper secondary education; ^b^High education: at least a Bachelor’s degree or equivalent level

As education is a major determinant of health behaviours, and especially of smoking [23], we considered the highest level of education as a control variable. For comparative purposes across countries, education was categorized in four levels, based on national typologies using ISCED (International Standard Classification of Education) standards [24]: 1) ISCED 0, 1 and 2 levels: below upper secondary education (Low); 2) ISCED 3 and 4: upper secondary education and post-secondary non tertiary education (Medium); 3) ISCED 5: short-cycle tertiary education (High-short); 4) ISCED 6 and over: at least a Bachelor’s degree or equivalent level (High-long). The educational gradient in smoking prevalence was quantified using the relative index of inequality [25, 26], that allow comparisons of populations with different education levels and age, taking into account the change in values of highest level of education across time and place. The ridit, a rank variable of our 4-category variable of education for each gender [27], was computed for this purpose. The relative index of inequality (RII) is the odds-ratio of the ridit, which contrasts the risk of smoking of the least to the most educated in the population, taking into account the distribution of education in the population. RII estimates lower than 1 correspond to higher prevalence among the most educated (positive gradient), whereas RII estimates greater than 1 correspond to lower prevalence among the most educated (negative gradient).

### Statistical analyses

Multivariate dichotomous logistic regressions modelling the smoking status at the time of the survey were run, adjusting for group of origin, ridit, age and age-squared. The regressions were conducted separately for men and women, except for the estimation of the male-to-female prevalence ratio. All analyses were conducted with SAS 9.4; the sampling design and survey weights were taken into account with the SURVEY procedures.

## Results

### Sample description

The different groups differed widely in their sociodemographic breakdown and smoking prevalence levels (Table 1).

#### Maghrebins

Immigrants from this group had a median age of 40 years at the time of the survey and a large proportion had arrived as adults (median ages at arrival of 20 years for men and 18 years for women, with median durations of stay of 20 and 24 years respectively). They were definitely less educated than the reference group, with an even greater disadvantage for women than for men (52% with low education vs 41%). Considering daily smoking, there was for this group a very sharp gender pattern, with a higher prevalence in men in comparison with the reference group (41% vs 33%) and a lower prevalence in women (14% vs 27%).

#### Repatriates from North Africa

Those immigrants were the oldest (median ages of 57 and 58 years for men and women respectively, as opposed to 43 and 44 years for the reference group), and those who had been settled in metropolitan France for the longest time (median age at arrival of 9–10 years with a median duration of 48 years for both genders). While both genders had a higher proportion of lower-educated than the reference group (36% vs 24% for men and 38% vs 29% for women), men had also a higher proportion of higher-educated (20% vs 14%). As a whole, prevalence of daily smoking among repatriates was close to that of the reference group for men (34% vs 33%), and lower for women (22% vs 27%).

#### Migrants from sub-Saharan Africa and from overseas '*départements*'

Those two groups were the youngest (median ages in the mid to late thirties), and while both had a median age at arrival around 20 years, the former arrived at a later time (median duration of stay of 15 and 13 years) than the latter (median duration of stay of 24 and 20 years). Male immigrants from overseas *départements* had a disadvantaged educational profile, while women from the same group were more concentrated in the intermediate categories. The presence among immigrants from sub-Saharan Africa of relatively greater (or equal) proportions in both the highest and lowest education categories reflects the heterogeneity of this group, with a mixture of labor migration and student migration. Both immigrants from overseas *départements* and immigrants from sub-Saharan Africa are characterized by a particularly low prevalence of daily smoking (around 20% or less).

### Patterns of smoking

#### Prevalence levels

The multivariate analysis provided a comprehensive description of the smoking patterns with special attention to the prevalence levels and the gender and social patterning of the different groups, taken as clues to their positioning within the cigarette epidemic course. After adjusting for age and relative educational level (ridit), there were significant variations in the proportion of daily smokers across migrant groups (Table 2).

**Table 2:** Odds ratio, relative index of inequality (RII) and male-to-female odds ratio for daily smoking in the different groups (in bold: significant at the 5% level at least; underlined: borderline significance (10% level))

	Men (*N* = 8407)			Women (*N* = 10,356)			Male-to-female odds ratio	
	Odds ratio^(a)^ (95% CI)	RII^(b)^		Odds ratio^(a)^ (95% CI)	RII^(b)^			
		Group-specific RII (95% CI)	Comparison of group-specific RII with reference population RII (95% CI)		Group-specific RII (95% CI)	Comparison of group-specific RII with reference population RII (95% CI)	Group-specific male-to-female odds ratio (95% CI)	Comparison of group-specific male-to-female odds ratio with reference population male-to-female odds ratio (95% CI)
Reference group	1.00	**3.64** (3.02–4.39)	1.00	1.00	**3.43** (2.84–4.15)		**1.33** (1.24–1.42)	1.00
Migrants group								
Maghrebins	1.23 (0.99–1.54)	**3.01** (1.34–6.79)	0.83(0.36–1.91)	**0.30** (0.21–0.41)	0.42 (0.13–1.29)	**0.12** (0.04–0.38)	**5.48** (3.69–8.15)	**4.14** (2.77–6.18)
Repatriates from north Africa	**1.82** (1.29–2.57)	1.38 (0.43–4.43)	0.38 (0.12–1.24)	1.44 (0.97–2.12)	3.44 (0.81–14.53)	1.00 (0.23–4.28)	**1.71** (1.03–2.85)	1.29 (0.77–2.16)
Migrants from sub-Saharan Africa	**0.41** (0.28–0.60)	**8.49** (2.10–34.37)	2.33 (0.57–9.56)	**0.42** (0.30–0.59)	**4.79** (1.19–19.25)	1.39 (0.34–5.68)	1.32 (0.80–2.18)	0.99 (0.60–1.65)
Migrants from french overseas *départements*	**0.43** (0.29–0.62)	1.15 (0.25–5.37)	0.32 (0.07–1.49)	**0.40** (0.26–0.62)	2.61 (0.37–18.33)	0.76 (0.11–5.39)	1.43 (0.81–2.54)	1.08 (0.61–0.93)

^a^Adjusted on age and education

^b^Relative index of inequality

Example: the adjusted odds ratio for smoking in the group of Maghrebin men is 1.23. In this group of men, the educational gradient is reflected by a RII of 3.0. Among Maghrebins, the male-to-female odds ratio is 5.48. This ratio is 4.14 times higher than that of the reference group, which is 1.33

Immigrants from sub-Saharan Africa and those from overseas *départements* had a much lower prevalence of daily smoking than the reference group (OR around 0.40, *p* < 0.001 for both genders). The only group reporting a significantly higher prevalence was that of male repatriates from North Africa (OR = 1.82, p < 0.001). The estimate for women in this group was relatively elevated, as was that of Maghrebin men (borderline significance for both). Maghrebin women had the lowest prevalence of all groups (OR = 0.30, *p* < 0.0001).

#### Male-to-female odds ratios

In the reference population, the prevalence in men was significantly higher than the prevalence in women (odds ratio = 1.33, *p* < 0.001). Repatriates, immigrants from overseas France and immigrants from sub-Saharan Africa had a men-to-women ratio comparable to that of natives (albeit not significantly different from 1 for the latter two groups). The gender ratio among Maghrebins was the most elevated of all groups (odds ratio = 5.48, *p* < 0.001).

#### Educational gradients in smoking

In the reference population, there was a significant pattern of higher prevalence among the least educated in both genders, i.e. a negative gradient (*Measures*), with a relative index of inequality (RII) estimate of 3.64 for men and 3.43 for women (*p* < 0.001 for both). This was also the case for male immigrants from Maghreb (RII estimate of 3.01, p < 0.001). This feature was extremely pronounced among immigrants from sub-Saharan Africa (RII estimate of 8.49, *p* < 0.01 for men; RII estimate of 4.79, *p* < 0.05 for women). The estimate for female Maghrebins reflected a somewhat higher prevalence among the most educated (0.42), while for females from overseas *départements* it reflected a higher prevalence among the least educated (2.61), but none of those patterns were significant, neither did they differ from the reference. Repatriated men from North Africa and those originating from overseas *départements* were found to have a relatively flat educational gradient (RII estimates of 1.38 and 1.15 respectively), with no significant difference in either case from the reference population.

## Discussion

Our findings illustrate the heterogeneity of migrant populations originating from three subsets of emigration countries, and question the generalizability of the notions of smoking transition and convergence with host population after immigration. The joint consideration of prevalence levels, men-to-women odds ratios and educational gradients in smoking (RII) with a comparative vision between immigrants and the reference population carries precious information with respect to the cigarette epidemic dynamics. Indeed, a large estimate for the relative index of inequality (RII) may be interpreted as reflecting a late stage, and similarly the closer to unity the male-to-female odds ratio, the later the stage.

### Historical aspects

The migrant groups in France are quite diverse in terms of their region of origin, history and circumstances of migration. The older waves were from the south of Europe, with migrants from Italy and Spain preceding migrants from Portugal, and both groups currently shrinking due to deaths and return migration. Migration from Algeria started to develop in the fifties and continued for several decades, while migration from either Morocco or Tunisia is more recent, and from sub-Saharan African countries even more.

Another important stream of migration to metropolitan France was that of European colonists coming in large numbers from the former French colonies in North Africa (the so-called *pieds-noirs* or repatriates). The great majority of those repatriates arrived from Algeria in the early 1960s, and quite a few have since died. A large part of those still alive in 2010 arrived in the metropole as children or adolescents accompanying their parents, as reflected in their early age at arrival and long duration of stay in metropolitan France. Repatriates were socially distinct from the low-wage laborers (Maghrebins) coming from the same country, as they had higher educational credentials and socioeconomic positions [22].

The population from French overseas *départements* (Guadeloupe, Martinique, French Guiana, Reunion), which represent the remainder of the past colonial empire, is predominantly Afro-Caribbean and Creole, and on the whole socio-economically disadvantaged. A majority of immigrants from overseas *départments* came during the past decades as adults to pursue their studies or integrate the work force [28].

This population history and composition is of particular interest for the study of smoking disparities and transitions. Most of the foreign-born immigrants originate from countries lagging behind metropolitan France in the course of the cigarette epidemic,^1^ as north African countries were positioned in the years 2000 in stage 2 and sub-Saharan countries in stage 1 [1]. As far as overseas *départements* are concerned, there is recent evidence of a low prevalence of cigarette smoking in Guadeloupe, Guyane and Martinique (< 20%), suggesting that those regions could be considered as belonging to stage 1, whereas the prevalence in La Reunion is relatively close to that of metropolitan France [29, 30].

### Group profiles

#### Maghrebins

Maghrebins stand out as the only group of immigrants with a strong gender discordance, as men have higher and women lower prevalence than natives. The particularly low prevalence of Maghrebin women, coupled with their slightly positive and nonsignificant gradient suggests that they may be positioned at a very early cigarette epidemic stage, as opposed to Maghrebin men, whose somewhat greater prevalence (borderline significance), possibly resulting in part from their exposure to particular hardship and disadvantaged profile, and reversed gradient are suggestive of a more advanced stage. As the smoking prevalence ratio was found to be strongly correlated worldwide with the gender empowerment measure [31], the impressive gap found in this study supports the idea of the persistence of traditional gender norms long after immigration. This gender pattern is also compatible with the pursuit post-migration of gender-specific epidemics, as hypothesized in a revision of the initial cigarette epidemic model [19].

Our finding of a higher smoking prevalence among Maghrebin men accords with reports from earlier studies based on french national health surveys from the late eighties [12] and the early nineties [13]. The same type of pattern was reported for Turkish immigrants in Germany and the Netherlands [8]. In the former country, a progressive prevalence rise in women with increasing length of stay in Germany and a slight decrease in men [7] were observed, leading to a call from the authors in favour of tailored public health interventions to help those migrant women arriving from countries in earlier stages of the cigarette epidemic to refrain from taking up smoking [8].

In a literature review of studies on smoking in immigrants from non-western to western countries, Reiss et al. [6] highlight the common feature of high prevalence among less acculturated men and low prevalence among less acculturated women originating from non-western countries. According to Leung [32], this particular gender pattern leads to a situation where “immigrant men tend to experience healthy assimilation, whereas immigrant women tend to experience unhealthy assimilation”. In the USA, the gender differences in smoking were found to be smaller among immigrants than in their countries of origin, but larger than among the US-born [33].

#### Repatriates

Contrary to the Maghrebins, the repatriates necessarily developed their patterns of entry into smoking in metropolitan France, where they arrived early and have spent most of their life. And yet, they have a distinctive smoking pattern, as men have a significantly higher prevalence than the reference population, and women tend to have higher prevalence (borderline significance). Another feature is the absence of a significantly reversed educational gradient in men only, whereas women have the same type of negative gradient as the reference population.

To our knowledge, the only comparable study in the literature concerns German ethnic immigrants from the former Soviet Union [9], who, unlike the repatriates in our study, immigrated while adults. The authors found that the male resettlers had higher smoking prevalence than the reference population at the time of their arrival in Germany, and women resettlers had lower smoking prevalence, but that both genders converged with the local levels with increasing duration of stay.

#### Migrants from sub-Saharan Africa

The group of immigrants from sub-Saharan Africa is very different from the two groups from North Africa in that both genders have lower prevalence than the natives. Those low prevalence levels reflect the situation in their countries of origin, as sub-Saharan countries are considered to be in the first stage of the cigarette epidemic. Sub-Saharan immigrants however have a distinctive pattern of sharply reversed educational gradient for both genders. Another striking feature is that the male-to-female odds ratio in sub-Saharan immigrants is relatively close to that of the reference population, as opposed to a much larger gap in their origin countries in sub-Saharan Africa [34]. Of all the foreign-born migrant groups, sub-Saharan immigrants are those with the highest proportion of higher educated, as quite a few in this group came to France to pursue their studies [35]. The combination of relatively balanced male-to-female ratio and definitely reversed educational gradient suggests that this group has reached an advanced stage of the cigarette epidemic.

#### Migrants from overseas '*départements*'

There was no evidence for immigrants from overseas *départements* of the high levels which could be expected based on their disadvantaged profile. Their relative protection from smoking in comparison with the reference population brings them close to the group from sub-Saharan Africa, and is more in line with the situation in their regions of origin [29, 30]. Further to that, their male-to-female prevalence ratio is comparable to that of the reference population in France, while the gender lag is much greater in overseas *départements* [30], which are still at the start of the cigarette epidemic. Finally, although their educational gradient is negative, more so for women than for men, neither estimate reaches significance, which suggests that the inversion of the gradient characteristic of the last phase of the cigarette epidemic has not yet taken place in this group.

### Enduring influence of region of origin situation at time of migration

The persistence of **low levels of smoking post-migration from stage 1 countries or regions** (sub-Saharan Africa, french overseas *départements*) may be related to the hypothesis that the so-called healthy migrant effect includes healthy behaviour [2, 4, 33]. Furthermore, the two groups concerned have the same men-to-women prevalence ratio as the natives, but they differ in the strength of their educational gradient. As sub-Saharan are of all immigrants, those with the shortest durations of stay, their very strong inversed pattern may imply that they have experienced an accelerated transition. At the opposite, the absence of a significantly reversed gradient among men from overseas *départements* and territories is indicative of an early stage and may raise concerns about future rises in prevalence.

**Emigrating from a stage 2 region** (North Africa) is associated with higher smoking prevalence in men, even on the long-term, and even when arriving at early ages (repatriates). The groups concerned did not have clearly reversed gradients, and this was particularly noteworthy for the long-standing residents who had developed their patterns of entry into smoking in metropolitan France after their arrival at an early age (repatriates). A **delay or maintenance of the smoking patterns** which the group had at the time of migration may be envisaged, along with the hypothesis of a kind of smoking socialization related to strongly anchored familial norms and habits. At the opposite, in the USA, the protective effect of immigrant status against smoking was interpreted in relation with an “anti-smoking socialization in families” [2, 33].

Maghrebins were the only group for which **genders had discordant relative positions.** The relative over-consumption of Maghrebin men is consistent with reports based on earlier surveys [12, 13]. The opposition between Maghrebin men and Maghrebin women, the latter being very much protected from smoking, is indicative of the persisting **influence of gender norms long post-migration in certain groups,** but not in others. Indeed, the gender gap in sub-Saharan Africa and in overseas *départements* is not reflected in the immigrants in metropolitan France. Furthermore, the absence of a significant inverse gradient in Maghrebin women may suggest that there is in this group the potential for a rise in the future.

### Policy implications

In their “operant model of acculturation”, Landrine and Klonoff [36] emphasize that “recent immigrants (and culturally traditional minorities) who are at risk are not those who exhibit health-damaging behaviors but rather, those who do not”. Tailored interventions to prevent smoking uptake (“unhealthy assimilation”) and maintain the prevalence of this health-damaging habit at low levels would be very useful for men and women from overseas *départements* as well as for Maghrebin women. Men and women from sub-Saharan Africa may also be targeted, although their very strong inverse gradient suggests that they may have reached the last stage of the epidemic with a stabilization of the prevalence at low levels. Smoking cessation efforts should also be encouraged specifically in groups characterized by high smoking levels, such as Maghrebin men. This could be achieved by offering tailored quit interventions or more culturally relevant programs [37] or alternatively through more inclusive approaches [38].

### Limitations

Regarding the analysis of the educational gradient in immigrants, a major difficulty is that educational categories do not have the same meaning depending on the national context, and this affects comparability between immigrants, who have been socialized and have attended school in their countries of origin, and natives [10]. One asset of our study is the use of a relative educational level, leading to relative indexes of inequality which can more safely be compared across groups as a measure of smoking gradient.

Lastly, the major limitation is that the study is based on cross-sectional analyses and therefore cannot provide a vision of the past dynamics in the different groups. Acculturation is a complex process unfolding over time, and while the patterns which we observed have lead us to generate hypotheses regarding the varying smoking transitions in the different groups, those need to be further investigated through longitudinal analyses in order to confirm the role of migration as a turning point in the transition processes.

## Conclusion

By considering migrant groups from different regions of origin and with different historical contexts of migration to metropolitan France and analysing their smoking habits through a multifaceted analytical approach, this study sheds light on very diverse smoking patterns, all distinct from those of the reference population. Some groups may have experienced an accelerated smoking transition, whereas others, some of which long-standing residents, may have stalled. The situation of Maghrebins, which stand out as the group with the most pronounced gender discordance, reflects the persistence of gendered norms post-migration. The absence of an inversed educational gradient in immigrants from overseas *départements* (particularly men) and in Maghrebin women, may suggest that those groups are positioned at an early stage of the cigarette epidemic, with a potential increase in the future of their strikingly low prevalence. Conversely, the very strong inversed pattern in immigrants from sub-Saharan Africa may suggest that they have experienced an accelerated transition. In that case, they are likely to retain their low levels of consumption. Repatriate men, who are long-standing residents, have both an elevated prevalence and an absence of significantly reversed gradient, which may indicate that they have remained at an early cigarette epidemic stage.

This comprehensive description provides some ground to foresee possible evolutions and recommend policy orientations. Tailored interventions to prevent *unhealthy assimilation* in groups still protected from smoking are advisable, particularly in Maghrebin women and in men and women from overseas *départements* and territories. Special efforts should also be made to reach Maghrebin men, who are exposed to specific risks related to their exposure to both socioeconomic disadvantage and hardship in the workplace.

## Footnotes

1 The cigarette epidemic model, as elaborated by Lopez and colleagues, comprises 4 stages [17, 18]. During stage 1, the prevalence of cigarette smoking is still lower than 20% and diseases and deaths related to smoking are not yet visible. Stage 2 is characterized by a rapid rise of smoking prevalence in men, the beginning of the rise in women, and the growth of smoking-related mortality. In stage 3, there is a flattening and downturn of the prevalence in men, leading to some convergence with the prevalence in women, while smoking attributable mortality rises to about 30% of all deaths, most of these in men. In the fourth and final stage, the prevalence declines in both genders, while smoking-attributable mortality continues to rise.
